# Benzol Treatment of Leukaemia

**Published:** 1914-06

**Authors:** 


**Benzol Treatment of Leukaemia.** L. F. Barker and James H. Gibbes, Bulletin of the Johns Hopkins Hospital, December, 1913; have collected the following from the literature: Splenomyelogenic type, 13 cases; lymphatic type, 3; (also, 1 each of pseudoleukaemia and polycythaemia with splenic tumor) and add a case of the first type. The treatment resulted in a fall from 120 to 345 thousand to 6 to 13 thousand whites. As is well known, benzol is not entirely free from danger and the results may not be permanent.

